# Real-world data on patient-reported outcomes and potential factors associated with pain after thyroid/parathyroid surgery: a prospective QUIPS registry analysis of a large cohort – sparking new pain management concepts

**DOI:** 10.1186/s12893-026-04101-w

**Published:** 2026-08-01

**Authors:** Lilit Flöther, Taras Shpyruk, Philipp Burow, Michael Bucher, Annett Christel, Rick Schneider, Kerstin Lorenz, Andreas Wienke, Lars Kurch, Winfried Meissner, Johannes Dreiling

**Affiliations:** 1 Department of Anesthesiology and Surgical Intensive Care Medicine, University Hospital Halle (Saale), Martin Luther University Halle-Wittenberg, 06120 Halle (Saale), Germany; 2 Department of Visceral, Vascular and Endocrine Surgery, University Hospital Halle (Saale), Martin Luther University Halle-Wittenberg, Halle, 06120 Germany; 3 Department of Nuclear Medicine, University Hospital Leipzig, University Leipzig, Leipzig, 04103 Germany; 4 Department of Anesthesiology and Intensive Care Medicine, University Hospital Jena, Friedrich Schiller University Jena, Jena, 07747 Germany; 5 Department of Palliative Care, University Hospital Jena, Friedrich Schiller University Jena, Jena, 07747 Germany; 6 Institute of Medical Epidemiology, Biometry and Informatics, Medical Faculty, Martin Luther University Halle-Wittenberg, Halle, 06112 Germany

**Keywords:** Thyroid/Parathyroid surgery, QUIPS, Perioperative pain management, Patient reported outcomes, Real-world data

## Abstract

**Background:**

Although thyroid and parathyroid surgeries are common elective procedures in Germany, postoperative pain management remains unstandardized. This prospective study sought to quantify postoperative pain intensity after thyroid and parathyroid surgery and to examine factors associated with postoperative pain.

**Methods:**

We prospectively enrolled 217 adult inpatients undergoing non-oncologic thyroid or parathyroid surgery between September 2021 and September 2022 into the QUIPS (Quality Improvement in Postoperative Pain Management) registry. On postoperative day one (POD1), patient-reported outcomes were assessed using the standardized QUIPS questionnaire, including an 11-point numeric rating scale (NRS) ranging from 0 to 10. Demographic, perioperative, and treatment-related variables were summarized descriptively, and associations with postoperative outcomes were examined using univariable, unadjusted group comparisons.

**Results:**

Postoperative pain was reported by 96.8% of the patients, with a mean maximum NRS score of 4.89 ± 2.76 and a mean pain-on-exertion score of 3.88 ± 2.32. In the univariable, unadjusted analyses, higher maximum pain scores were observed among women, patients younger than 65 years, patients with pre-existing chronic pain, and patients classified as ASA I. Maximum pain scores differed according to the type of surgery and were highest after total thyroidectomy. Pain on exertion and pain-related mood impairment also differed across operative-duration groups, with the highest values observed after procedures lasting 121–180 min. Individualized preoperative information on postoperative pain management was associated with higher patient satisfaction and a lower reported desire for additional analgesia.

**Conclusion:**

Moderate pain was frequently reported after thyroid and parathyroid surgery, with pain intensity varying according to patient- and procedure-related characteristics in unadjusted analyses.

Our findings support the implementation of standardized approaches to postoperative pain management and preoperative patient education.

## Introduction

Benign thyroid disorders affect approximately 20–30% of the German population, while the prevalence of primary hyperthyroidism is around 1% [1, 2]. Approximately 100,000 thyroid surgeries are performed annually in Germany—four to six times more frequently than in many other countries [3]. Among patients with benign nodular thyroid disorders, approximately 50% undergo total thyroidectomy and around 40% hemithyroidectomy [4].

Approximately 10% of patients experience moderate to severe postoperative pain following thyroid surgery [5]. Persistent postoperative pain is recognized as a risk factor for the development of chronic pain, which can significantly impair quality of life [6, 7]. Effective perioperative pain management not only reduces postoperative pain but also contributes to reducing perioperative complications, shortens hospital stays, and lowers overall healthcare costs [8].

Procedure-specific postoperative pain management recommendations (PROSPECT) exist for many types of surgery [9]. However, such evidence-based guidelines are currently lacking for thyroid and parathyroid procedures.

The aim of this prospective study was to systematically analyze patient-reported outcomes following thyroid and parathyroid surgery and to examine possible associations between patient-, procedure-, and treatment-related characteristics as well as postoperative pain outcomes.

## Methods

### Patients

This prospective, non-interventional clinical observational trial was conducted at the Department of Anaesthesiology and Operative Intensive Care Medicine in collaboration with the Department of Visceral, Vascular and Endocrine Surgery at the University Hospital of Halle (Germany).

Ethical approval was obtained from the local ethics committee (identification number: 2021-094). 

All adult inpatients who underwent total thyroidectomy, hemi-thyroidectomy, partial para-thyroidectomy and para-thyroidectomy between September 2021 and September 2022 were enrolled.

Patients with preoperative evidence of malignancy and/or intended neck dissection were excluded in order to obtain a more homogeneous study population and to minimize potential confounding associated with oncologic procedures. In particular, neck dissections and more extensive resections may substantially influence postoperative pain intensity and recovery patterns compared with surgery for benign disease.

All study patients gave their written informed consent.

## Data

Data was collected within the QUIPS (Quality Improvement in Postoperative Pain Management) registry (German Clinical Trials Register ID: DRKS00006153). The QUIPS registry collects multidimensional patient-reported outcomes (PROs), demographic variables, and processes variables in a standardized format [8]. The registry aims to enhance the quality of early postoperative pain management by implementing standardized data collection, evaluating PROs and process indicators, and providing feedback and benchmarking to the participating study centres. PROs and process variables were collected in a standardized way with the QUIPS questionnaire on postoperative day 1 (POD1) [8].

We primarily analyzed the main variables of the patient questionnaire: pain intensity (maximum, minimum, and during activities, 0–10 on the numeric rating scale [NRS]), pain-related interference (during movement, coughing/deep breathing, sleep, and mood; yes versus no) and side effects (drowsiness and nausea; yes versus no). Demographic (age, sex, chronic pain status, American Society of Anesthesiologists (ASA) Classification) and process variables (e.g. type of anaesthesia, duration of surgery, postoperative pain management, and surgery specific complications) were extracted from the patient’s medical record.

Postoperative pain management was, however, organized by the ward responsible for further care.

### Statistics

The collected data were analyzed using SPSS version 29.0. Mean values and standard deviations were calculated for continuous variables. Chi-square tests were used for comparisons of categorical data. Continuous data were compared using the Wilcoxon–Mann–Whitney test. For comparisons involving more than two groups, analysis of variance (ANOVA) followed by Tukey’s honestly significant difference post hoc test was used. The significance level was set at *p* < 0.05. All analyses were univariable and unadjusted.

## Results

### Patient cohort

A total of 217 patients were enrolled, of whom 138 (63.6%) were female. The mean age was 55.6 ± 15.0 years, with a range from 19 to 85 years. Chronic pain conditions were reported by 38 patients (17.5%), five of whom were taking pain medication on a regular basis.

All surgeries were performed under general anesthesia. No additional local anesthetic was administered through wound margin infiltration. Total thyroidectomy was the most common procedure, accounting for 55.8% of cases (121/217). Nearly half of the procedures lasted between 61 and 120 min. Perioperative and postoperative antibiotic prophylaxis was not routinely administered. No postoperative bleeding or other serious complications were observed in any patient.

Further patient- and procedure-specific characteristics are summarized in Table 1.

**Table 1:** Patient- and procedure-specific characteristics of the study cohort

Patient- and procedure-specific characteristics (*n* = 217)		
Parameter	(*n*)	(%)
Age		
≥ 65 years	88	40.6
< 65 years	129	59.4
Sex		
male	79	36.4
female	138	63.6
ASA physical status		
I	11	5.1
II	152	70.0
III	54	24.9
Chronic pain prior to surgery		
yes	38	17.5
no	179	82.5
Operation type		
Total thyroidectomy OPS 5–063	121	55.8
Hemithyroidectomy / partial thyroidectomy OPS 5–061/5–062	60	27.6
(partial or total) OPS 5–066/5–067	36	16.6
Duration of surgery		
up to 60 min	33	15.2
61 to 120 min	104	47.9
121 to 180 min	54	24.9
Longer than 180 min	26	12.0

### QUIPS outcome parameters

**Fig. 1:**
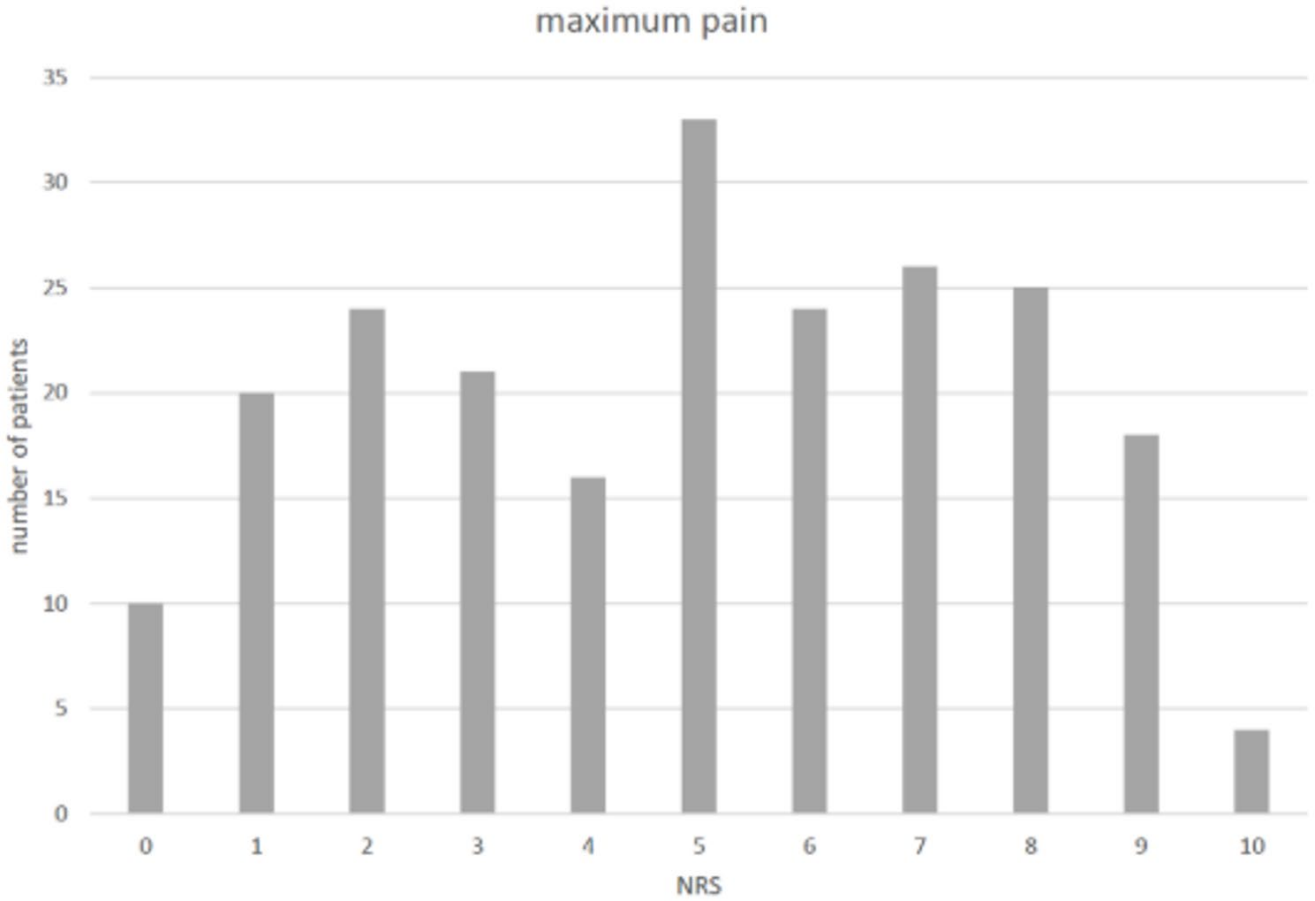
Distribution of patients with the strongest reported pain using the numerical rating scale (NRS); higher values indicate more pain (0-no pain, 10-maximum pain)

The results of the survey on postoperative pain are summarized in Table 2; Fig. 1.

**Table 2:** Outcome parameters from the QUIPS survey

Outcome parameters (*n* = 217)			
Parameter	mean ± SD	(*n*)	(%)
Pain on exertion (NRS)	3.88 ± 2.32		
Maximum pain (NRS)	4.89 ± 2.76		
Minimum pain (NRS)	1.88 ± 1.62		
Satisfaction with pain therapy (NRSᵃ)	7.64 ± 2.77		
Preoperative information on pain therapy	Yes	123	56.7
Pain-related mobility impairment	Yes	62	28.6
Pain-related respiratory impairment	Yes	123	56.7
Pain-related sleep impairment	Yes	80	36.9
Pain-related mood impairment	Yes	36	16.6
Desire for more treatment	Yes	33	15.2
Fatigue	Yes	114	52.5
Nausea	Yes	46	21.2
Vertigo	Yes	56	25.8

*NRS*  Numerical Rating Scale, *SD*  standard deviation

^a^ numerical 11-point scale, higher numbers indicate more patient satisfaction (0-no satisfaction, 10-maximum satisfaction)

The intensity of maximum postoperative pain varied considerably, ranging from no pain to mild, moderate, and severe levels (Fig. 1).

Both exertional pain and maximum pain were, on average, rated as moderate, with maximum pain scores being only slightly higher than exertional pain scores (maximum pain: 4.89 ± 2.76; pain on exertion: 3.88 ± 2.32). In the subgroup of patients with pre-existing chronic pain, the average maximum NRS score was 5.66, compared to 4.73 in patients without chronic pain.

A total of 123 patients (56.7%) received preoperative information regarding postoperative pain management.

Pain-related respiratory impairment was the most frequently reported adverse effect, affecting 123 patients (56.7%). Mobility restrictions due to pain were reported by 62 patients (28.6%), and 80 patients (36.9%) complained of pain-related sleep impairment. Pain-related mood impairment was mentioned by 36 patients (16.6%). Nausea was reported by 46 patients (21.2%), and 56 patients (25.8%) reported vertigo.

Overall satisfaction with pain management was rated at an average of 7.64 ± 2.77 (on a scale of 0 to 10, with 10 indicating highest satisfaction).

### QUIPS process parameters

An overview of the perioperative and postoperative measures used for pain prevention and management is provided in Table 3.

**Table 3:** Process parameters of the QUIPS survey

Parameter	Category	*n*	Proportion (%)
Anaesthesia procedure	General anaesthesia	217	100.0
Non-opioid analgesics (recovery room)	None	32	14.7
	Metamizole	163	75.1
	Paracetamol	8	3.7
	Metamizole + Paracetamol	14	6.5
Opioid analgesics (recovery room)	None	20	9.2
	Piritramide	74	34.1
	Oxycodone	123	56.7
Analgesic treatment on the ward	None	175	80.6
	Non-opioids	35	16.1
	Opioids	7	3.2
Non-pharmacological interventions on the ward	Yes	66	30.4
Pain therapy instructions on the ward	Yes	69	31.8
Standardized pain documentation on the ward	Yes	217	100.0

All patients received sufentanil intraoperatively as part of general anesthesia. Ketamine was not used in any patient. In the recovery room, metamizole alone was administered to 163 patients (75.1%), paracetamol alone to 8 patients (3.7%), and a combination of metamizole and paracetamol to 14 patients (6.5%). A total of 32 patients (14.7%) received no non-opioid analgesic.

Intravenous oxycodone was administered to 123 patients (56.7%) and piritramide to 74 patients (34.1%), while 20 patients (9.2%) received no opioid in the recovery room.

Following transfer to the ward, 175 patients (80.6%) received no analgesic medication. Non-opioids were administered to 35 patients (16.1%), while 7 patients (3.2%) received opioids. A total of 69 patients (31.8%) had either an on-demand or a fixed-dose analgesic regimen prescribed. Pain intensity was recorded for all patients at eight-hour intervals.

### Univariable associations between patient-, procedure-, and treatment-related characteristics and outcomes

The results from the univariable analyses of process and outcome parameters are shown in Tables 4, 5, 6 and 7.

**Table 4:** Associations of patient and procedure characteristics with postoperative outcomes Continuous outcomes, mean ± SD

Characteristic	Group	Pain on exertion NRS 0–10	Maximum pain NRS 0–10	Minimum pain NRS 0–10	Satisfaction with pain therapy NRS 0–10
Sex	Female	4.08 ± 2.41	5.25 ± 2.91	2.06 ± 1.75	7.43 ± 3.03
	Male	3.54 ± 2.16	4.27 ± 2.39	1.56 ± 1.34	8.01 ± 2.23
p-value		0.115	0.007	0.065	0.411
Age	< 65 years	4.10 ± 2.18	5.27 ± 2.67	2.00 ± 1.57	7.55 ± 2.76
	≥ 65 years	3.57 ± 2.51	4.34 ± 2.83	1.69 ± 1.70	7.77 ± 2.79
p-value		0.083	0.016	0.074	0.325
ASA	I	4.64 ± 2.20	6.00 ± 2.49	2.18 ± 0.98	7.45 ± 2.54
	II	4.10 ± 2.30	5.22 ± 2.64	1.94 ± 1.62	7.65 ± 2.76
	III	3.13 ± 2.31	3.74 ± 2.86	1.63 ± 1.73	7.65 ± 2.89
p-value		0.017	0.001	0.393	0.974
Surgery type	Hemithyroidectomy	3.78 ± 2.23	5.17 ± 2.74	1.72 ± 1.34	7.70 ± 2.64
	Total thyroidectomy	4.38 ± 2.27	5.33 ± 2.60	2.19 ± 1.72	7.60 ± 2.64
	Parathyroidectomy	2.39 ± 2.06	2.97 ± 2.62	1.08 ± 1.42	7.67 ± 3.42
p-value		< 0.001	< 0.001	< 0.001	0.974
Duration of surgery	≤ 60 min	3.67 ± 2.71	4.79 ± 2.87	1.58 ± 1.77	7.70 ± 3.37
	61–120 min	3.58 ± 2.18	4.63 ± 2.79	1.72 ± 1.32	7.34 ± 2.88
	121–180 min	4.70 ± 2.30	5.48 ± 2.63	2.50 ± 1.90	7.85 ± 2.44
	> 180 min	3.69 ± 2.19	4.85 ± 2.80	1.58 ± 1.68	8.35 ± 2.00
p-value		0.029	0.335	0.011	0.355
Chronic pain	No	3.75 ± 2.27	4.73 ± 2.68	1.80 ± 1.53	7.80 ± 2.61
	Yes	4.50 ± 2.53	5.66 ± 3.07	2.24 ± 2.01	6.87 ± 3.36
p-value		0.073	0.045	0.307	0.151

**Table 5:** Binary outcomes, n (%) with outcome present

Characteristic	Group	Pain-related sleep impairment	Pain-related mobility impairment	Pain-related respiratory impairment	Pain-related mood impairment	Fatigue	Vertigo	Nausea	Desire for more treatment
Sex	Female	59 (42.8)	46 (33.3)	83 (60.1)	27 (19.6)	88 (63.8)	47 (34.1)	37 (26.8)	24 (17.4)
	Male	21 (26.6)	16 (20.3)	40 (50.6)	9 (11.4)	26 (32.9)	9 (11.4)	9 (11.4)	9 (11.4)
p-value		0.018	0.040	0.174	0.119	< 0.001	< 0.001	0.007	0.236
Age	< 65 years	53 (41.1)	43 (33.3)	82 (63.6)	25 (19.4)	78 (60.5)	37 (28.7)	33 (25.6)	23 (17.8)
	≥ 65 years	27 (30.7)	19 (21.6)	41 (46.6)	11 (12.5)	36 (40.9)	19 (21.6)	13 (14.8)	10 (11.4)
p-value		0.119	0.060	0.013	0.181	0.005	0.241	0.056	0.193
ASA	I	2 (18.2)	5 (45.5)	6 (54.5)	2 (18.2)	5 (45.5)	5 (45.5)	5 (45.5)	2 (18.2)
	II	62 (40.8)	42 (27.6)	91 (59.9)	28 (18.4)	88 (57.9)	40 (26.3)	36 (23.7)	26 (17.1)
	III	16 (29.6)	15 (27.8)	26 (48.1)	6 (11.1)	21 (38.9)	11 (20.4)	5 (9.3)	5 (9.3)
p-value		0.144	0.445	0.325	0.458	0.050	0.215	0.011	0.371
Surgery type	Hemithyroidectomy	23 (38.3)	17 (28.3)	35 (58.3)	10 (16.7)	32 (53.3)	17 (28.3)	14 (23.3)	7 (11.7)
	Total thyroidectomy	49 (40.5)	41 (33.9)	74 (61.2)	22 (18.2)	69 (57.0)	31 (25.6)	26 (21.5)	22 (18.2)
	Parathyroidectomy	8 (22.2)	4 (11.1)	14 (38.9)	4 (11.1)	13 (36.1)	8 (22.2)	6 (16.7)	4 (11.1)
p-value		0.132	0.029	0.058	0.606	0.087	0.801	0.736	0.390
Duration of surgery	≤ 60 min	12 (36.4)	9 (27.3)	17 (51.5)	2 (6.1)	16 (48.5)	12 (36.4)	8 (24.2)	5 (15.2)
	61–120 min	37 (35.6)	24 (23.1)	61 (58.7)	13 (12.5)	53 (51.0)	24 (23.1)	22 (21.2)	15 (14.4)
	121–180 min	23 (42.6)	21 (38.9)	32 (59.3)	15 (27.8)	31 (57.4)	14 (25.9)	10 (18.5)	8 (14.8)
	> 180 min	8 (30.8)	8 (30.8)	13 (50.0)	6 (23.1)	14 (53.8)	6 (23.1)	6 (23.1)	5 (19.2)
p-value		0.740	0.217	0.767	0.023	0.837	0.489	0.925	0.944
Chronic pain	No	56 (31.3)	44 (24.6)	98 (54.7)	23 (12.8)	90 (50.3)	41 (22.9)	35 (19.6)	26 (14.5)
	Yes	24 (63.2)	18 (47.4)	25 (65.8)	13 (34.2)	24 (63.2)	15 (39.5)	11 (28.9)	7 (18.4)
p-value		< 0.001	0.005	0.212	0.001	0.149	0.034	0.198	0.544

*NRS* Numerical Rating Scale, *SD* Standard deviation. Two-group comparisons of continuous outcomes used the Mann–Whitney U test; comparisons involving more than two groups used one-way ANOVA. Binary outcomes were compared using Pearson’s chi-square test. All percentages use the respective group as denominator

**Table 6:** Associations of perioperative process variables with postoperative outcomes Continuous outcomes, mean ± SD

Process variable	Group	Pain on exertion NRS 0–10	Maximum pain NRS 0–10	Minimum pain NRS 0–10	Satisfaction with pain therapy NRS 0–10
Preoperative information on pain therapy	No	3.79 ± 2.28	4.95 ± 2.86	1.83 ± 1.45	6.64 ± 3.07
	Yes	3.96 ± 2.37	4.85 ± 2.70	1.91 ± 1.75	8.41 ± 2.25
p-value		0.603	0.767	0.891	< 0.001
Non-opioids in recovery room	Not given	3.22 ± 2.01	4.06 ± 2.66	1.53 ± 1.24	8.78 ± 1.54
	Metamizole	4.04 ± 2.35	5.02 ± 2.71	1.93 ± 1.70	7.48 ± 2.82
	Paracetamol	4.00 ± 2.51	5.75 ± 3.15	1.75 ± 1.16	7.25 ± 3.45
	Metamizole + paracetamol	3.50 ± 2.62	4.79 ± 3.31	2.07 ± 1.69	7.07 ± 3.56
p-value		0.289	0.258	0.598	0.082
Opioids in recovery room	Not given	2.50 ± 2.12	3.10 ± 2.69	1.20 ± 1.11	9.40 ± 0.99
	Piritramide	3.86 ± 2.19	5.00 ± 2.64	1.92 ± 1.63	7.07 ± 3.24
	Oxycodone	4.12 ± 2.38	5.12 ± 2.77	1.96 ± 1.68	7.70 ± 2.54
p-value		0.015	0.009	0.147	0.003
Non-pharmacological interventions on ward	No	3.77 ± 2.28	4.72 ± 2.77	1.84 ± 1.58	7.59 ± 2.91
	Yes	4.14 ± 2.43	5.29 ± 2.73	1.95 ± 1.74	7.76 ± 2.46
p-value		0.306	0.141	0.800	0.706
Pain therapy instructions on ward	No	3.75 ± 2.31	4.76 ± 2.69	1.69 ± 1.49	7.82 ± 2.73
	Yes	4.17 ± 2.37	5.19 ± 2.93	2.28 ± 1.82	7.25 ± 2.85
p-value		0.196	0.297	0.027	0.072
Pain therapy on ward	Not given	3.74 ± 2.25	4.78 ± 2.68	1.74 ± 1.52	7.70 ± 2.72
	Non-opioids	4.91 ± 2.41	5.91 ± 2.81	2.60 ± 1.85	7.89 ± 2.45
	Opioids	2.29 ± 2.56	2.57 ± 3.21	1.57 ± 2.15	5.00 ± 4.43
p-value		0.004	0.006	0.015	0.034

**Table 7:** Binary outcomes, n (%) with outcome present

Process variable	Group	Pain-related sleep impairment	Pain-related mobility impairment	Pain-related respiratory impairment	Pain-related mood impairment	Fatigue	Vertigo	Nausea	Desire for more treatment
Preoperative information on pain therapy	No	36 (38.3)	26 (27.7)	53 (56.4)	14 (14.9)	51 (54.3)	23 (24.5)	18 (19.1)	27 (28.7)
	Yes	44 (35.8)	36 (29.3)	70 (56.9)	22 (17.9)	63 (51.2)	33 (26.8)	28 (22.8)	6 (4.9)
p-value		0.702	0.795	0.938	0.557	0.657	0.694	0.518	< 0.001
Non-opioids in recovery room	Not given	10 (31.2)	7 (21.9)	14 (43.8)	1 (3.1)	15 (46.9)	8 (25.0)	8 (25.0)	3 (9.4)
	Metamizole	60 (36.8)	49 (30.1)	97 (59.5)	28 (17.2)	85 (52.1)	38 (23.3)	31 (19.0)	25 (15.3)
	Paracetamol	4 (50.0)	3 (37.5)	3 (37.5)	3 (37.5)	7 (87.5)	5 (62.5)	4 (50.0)	2 (25.0)
	Metamizole + paracetamol	6 (42.9)	3 (21.4)	9 (64.3)	4 (28.6)	7 (50.0)	5 (35.7)	3 (21.4)	3 (21.4)
p-value		0.743	0.672	0.237	0.042	0.223	0.076	0.194	0.602
Opioids in recovery room	Not given	5 (25.0)	4 (20.0)	9 (45.0)	1 (5.0)	9 (45.0)	2 (10.0)	5 (25.0)	1 (5.0)
	Piritramide	22 (29.7)	19 (25.7)	43 (58.1)	13 (17.6)	32 (43.2)	20 (27.0)	15 (20.3)	14 (18.9)
	Oxycodone	53 (43.1)	39 (31.7)	71 (57.7)	22 (17.9)	73 (59.3)	34 (27.6)	26 (21.1)	18 (14.6)
p-value		0.087	0.446	0.541	0.343	0.070	0.236	0.900	0.296
Non-pharmacological interventions on ward	No	46 (30.5)	40 (26.5)	80 (53.0)	21 (13.9)	75 (49.7)	28 (18.5)	26 (17.2)	20 (13.2)
	Yes	34 (51.5)	22 (33.3)	43 (65.2)	15 (22.7)	39 (59.1)	28 (42.4)	20 (30.3)	13 (19.7)
p-value		0.003	0.305	0.096	0.108	0.201	< 0.001	0.030	0.223
Pain therapy instructions on ward	No	50 (33.8)	40 (27.0)	79 (53.4)	19 (12.8)	77 (52.0)	38 (25.7)	35 (23.6)	17 (11.5)
	Yes	30 (43.5)	22 (31.9)	44 (63.8)	17 (24.6)	37 (53.6)	18 (26.1)	11 (15.9)	16 (23.2)
p-value		0.168	0.461	0.150	0.030	0.826	0.949	0.196	0.025
Pain therapy on ward	Not given	62 (35.4)	47 (26.9)	97 (55.4)	28 (16.0)	92 (52.6)	45 (25.7)	37 (21.1)	21 (12.0)
	Non-opioids	17 (48.6)	13 (37.1)	25 (71.4)	8 (22.9)	20 (57.1)	11 (31.4)	9 (25.7)	11 (31.4)
	Opioids	1 (14.3)	2 (28.6)	1 (14.3)	0 (0.0)	2 (28.6)	0 (0.0)	0 (0.0)	1 (14.3)
p-value		0.153	0.470	0.015	0.297	0.385	0.222	0.315	0.014

*NRS* Numerical Rating Scale, *SD* Standard deviation. Two-group comparisons of continuous outcomes used the Mann–Whitney U test; comparisons involving more than two groups used one-way ANOVA. Binary outcomes were compared using Pearson’s chi-square test. All percentages use the respective group as denominator

Sex was associated with postoperative pain intensity and related outcomes. Women reported higher maximum pain scores and more frequent pain-related sleep and mobility impairment. Nausea, vertigo, and fatigue were also more frequent among women. Age group was also associated with postoperative pain and related outcomes. Patients younger than 65 years reported higher maximum pain scores and more frequent pain-related respiratory impairment and fatigue. Regarding ASA classification, otherwise healthy patients classified as ASA I reported higher maximum pain scores and greater pain on exertion. Patients classified as ASA II reported nausea and fatigue more frequently.

Patients undergoing total thyroidectomy reported a mean maximum pain score of 5.33 ± 2.60, compared with 5.17 ± 2.74 after hemithyroidectomy and 2.97 ± 2.62 after parathyroidectomy (*p* < 0.001). Pain-related mobility impairment was reported by 33.9%, 28.3%, and 11.1% of patients, respectively (*p* = 0.029). Pain-on-exertion scores differed across operative-duration groups (*p* = 0.029), with the highest mean score observed in patients whose procedures lasted 121–180 min (4.70 ± 2.30). Pain-related mood impairment also differed across duration groups (*p* = 0.023) and was most frequent after procedures lasting 121–180 min (27.8%).

Patients with pre-existing chronic pain reported higher maximum pain scores and more frequent pain-related mobility, sleep, and mood impairment, as well as vertigo.

Patients who received individualized preoperative information on postoperative pain management reported higher satisfaction than those who did not receive such information (8.41 ± 2.25 vs. 6.64 ± 3.07; *p* < 0.001). They also reported a lower desire for additional analgesia (4.9% vs. 28.7%; *p* < 0.001).

On postoperative day 1 (POD1), pain on exertion differed according to opioid treatment in the recovery room, with mean scores of 2.50 ± 2.12 in patients receiving no opioid, 3.86 ± 2.19 in those receiving piritramide, and 4.12 ± 2.38 in those receiving oxycodone (*p* = 0.015). The corresponding maximum pain scores were 3.10 ± 2.69, 5.00 ± 2.64, and 5.12 ± 2.77, respectively (*p* = 0.009), while the mean satisfaction scores were 9.40 ± 0.99, 7.07 ± 3.24, and 7.70 ± 2.54, respectively (*p* = 0.003).

Patients who received instructions on pain and pain treatment on the ward (69/217, 31.8%) reported higher minimum pain scores than those without such instructions (2.28 ± 1.82 vs. 1.69 ± 1.49; *p* = 0.027). Pain-related mood impairment (24.6% vs. 12.8%; *p* = 0.030) and the desire for additional analgesia (23.2% vs. 11.5%; *p* = 0.025) were also reported more frequently.

Pain outcomes differed among patients receiving no analgesia, non-opioids, or opioids on the ward. Mean pain-on-exertion scores were 3.74 ± 2.25, 4.91 ± 2.41, and 2.29 ± 2.56, respectively (*p* = 0.004). The corresponding maximum pain scores were 4.78 ± 2.68, 5.91 ± 2.81, and 2.57 ± 3.21 (*p* = 0.006), and minimum pain scores were 1.74 ± 1.52, 2.60 ± 1.85, and 1.57 ± 2.15 (*p* = 0.015). Satisfaction with pain therapy was 7.70 ± 2.72, 7.89 ± 2.45, and 5.00 ± 4.43, respectively (*p* = 0.034). Pain-related respiratory impairment was reported by 55.4%, 71.4%, and 14.3% of patients (*p* = 0.015), while the desire for additional analgesia was reported by 12.0%, 31.4%, and 14.3%, respectively (*p* = 0.014). The opioid group comprised seven patients.

Patients who received non-pharmacological interventions on the ward reported more frequent pain-related sleep impairment (51.5% vs. 30.5%; *p* = 0.003), vertigo (42.4% vs. 18.5%; *p* < 0.001), and nausea (30.3% vs. 17.2%; *p* = 0.030) than those who did not receive such interventions.

## Discussion

In this prospective observational study, postoperative pain intensity, pain management, and associations with patient-, procedure-, and treatment-related characteristics were evaluated in 217 patients undergoing total thyroidectomy, hemithyroidectomy, or parathyroidectomy using the standardized QUIPS assessment tool. To the best of our knowledge, this cohort represents the largest prospective analysis to date focusing on postoperative pain in patients following thyroid or parathyroid surgery.

Over 96% of patients reported postoperative pain, with approximately 75% rating their pain intensity above 3 on the NRS, underscoring the clinical importance of effective pain management in this population. Particularly notable are the findings related to peak pain and pain during movement. Patients undergoing thyroid or parathyroid surgery reported moderate pain levels, with a mean maximum pain score of 4.89 and a mean pain-on-exertion score of 3.88 on the NRS. These pain intensities align closely with previous research, which has documented comparable peak pain levels ranging between 4 and 6 on the NRS after thyroid and parathyroid surgeries [10]. However, postoperative pain management is not mentioned in the procedure-specific guidelines for endocrine surgery. Even the recently published evidence-based guideline on the management of acute perioperative and post-traumatic pain does not account for the specific characteristics of different types of thyroid surgery necessary to adequately address procedure-specific pain management needs [11, 12].

Postoperative pain outcomes differed according to the type of surgery. Mean maximum pain scores were 5.33 after total thyroidectomy, 5.17 after hemithyroidectomy, and 2.97 after parathyroidectomy. Pain-related mobility impairment also differed across the three procedure groups. Pain on exertion and pain-related mood impairment differed across operative-duration groups, with the highest values observed after procedures lasting 121–180 min. As these analyses were unadjusted, they do not establish an independent effect of surgical extent or operative duration on postoperative pain. Nevertheless, previous studies have suggested that the extent of surgical trauma and the duration of the procedure may be relevant to postoperative pain intensity [13, 14].

In addition, patient positioning during surgery may also influence postoperative pain. The hyperextension of the neck required during thyroid surgery can lead to muscular strain or nerve irritation, particularly in the cervical and shoulder regions. Optimal intraoperative positioning and support (e.g., through soft padding and maintaining a neutral neck alignment) may therefore play a preventive role [15]. However, hyperextension of the neck was not assessed in our study, which represents a limitation that should be addressed in future research. Several recent studies suggest that bilateral superficial cervical plexus blocks can significantly reduce postoperative pain scores and opioid requirements after thyroidectomy [16, 17]. While this technique was not applied in our cohort, it represents a promising component of multimodal analgesia strategies and should be considered in future procedural guidelines.

Another finding of our research was the fact that maximum postoperative pain scores differed according to ASA classification, with the highest mean scores observed among patients classified as ASA I. This pattern is consistent with the findings of Wittekindt et al. [18], who reported higher postoperative pain among otherwise healthy patients with a lower ASA classification than among patients with greater comorbidity.

Furthermore, patients with pre-existing chronic pain reported higher peak pain levels, consistent with previous studies that highlighted increased pain sensitivity and impaired pain coping mechanisms within this population [19].

Our study findings reveal considerable variability in pain management on the postoperative ward compared to the recovery room, as evidenced by the differences in administered analgesics. While non-opioid analgesics were commonly administered in the recovery room, their use decreased substantially after patient transfer to the ward. This highlights the need for a standardized pain management protocol extending beyond the recovery room to ensure effective postoperative pain control until discharge. Such concepts have been proposed within Enhanced Recovery After Surgery (ERAS) pathways for thyroid and parathyroid surgery. Lide et al. demonstrated that structured perioperative care emphasizing multimodal analgesia, patient education, and reduced opioid exposure can be successfully implemented while maintaining adequate pain control [20]. Our findings support these concepts and suggest that further standardization of postoperative pain management may improve patient outcomes and reduce unwarranted variations in clinical practice.

In the unadjusted analyses, individualized preoperative information on postoperative pain management was associated with higher patient satisfaction and a lower reported desire for additional analgesia. Similar associations between preoperative information and patient-reported outcomes have been described in previous studies [18, 21].

Furthermore, standardized pain management pathways may contribute to a more efficient use of healthcare resources by reducing unnecessary treatment variation and improving patient recovery.

Apart from pain intensity, postoperative symptoms such as respiratory impairment, fatigue, nausea, and vertigo were frequently reported. Although often considered secondary outcomes, these symptoms may substantially affect recovery, mobilization, sleep quality, and patient satisfaction. Therefore, postoperative care following thyroid and parathyroid surgery should address not only pain intensity but also these associated symptoms.

Besides the previously mentioned limitation related to intraoperative patient positioning, several other limitations warrant consideration. Most importantly, all analyses were univariable and unadjusted. Therefore, the observed associations may be affected by confounding and cannot be interpreted as independent effects or causal relationships. Furthermore, multiple group comparisons were conducted without adjustment for multiple testing; consequently, the findings should be regarded as exploratory. The very small number of patients receiving opioids on the ward (*n* = 7) further limits the robustness and interpretation of comparisons involving this subgroup. The assessment of postoperative pain at a single time point on postoperative day 1 (POD1) does not allow for a comprehensive understanding of pain trajectories after hospital discharge. No conclusions can be drawn regarding long-term pain outcomes or recovery patterns.

From a methodological perspective, the exclusion of patients with preoperative evidence of thyroid malignancy limits the generalizability of our findings to oncologic thyroid surgery. Furthermore, detailed surgical variables, such as the indication for surgery, reoperative procedures, substernal extension, hyperthyroidism, or division of the strap muscles, were not available within the QUIPS registry, but could be of high value for broader multidisciplinary analyses. Surgical characteristics may influence postoperative pain outcomes and, thus, need to be evaluated in future studies. Operative duration was the only available indirect measure of procedural complexity. However, it cannot adequately capture differences in surgical indications or intraoperative characteristics. Lastly, healthcare costs related to postoperative pain management were not part of this analysis. The economic implications of different pain management approaches could therefore not be quantified. Thus, future studies should include economic evaluations to assess whether standardized pain management pathways may contribute to a more efficient use of healthcare resources, for example, by reducing unnecessary treatment variation or improving patient recovery.

## Conclusion

Patients undergoing thyroid or parathyroid surgery commonly experience acute postoperative pain.

In the unadjusted analyses, postoperative pain outcomes varied according to patient-, procedure-, and treatment-related characteristics. Maximum pain scores differed across procedure groups, with the lowest mean score observed after parathyroidectomy. Individualized preoperative information on postoperative pain management was associated with higher patient satisfaction and a lower reported desire for additional analgesia.

These findings support the implementation of standardized approaches to postoperative pain management and preoperative patient education in thyroid and parathyroid surgery.

## Data Availability

The datasets generated and/or analyzed during the current study are not publicly available due to data protection requirements but are available from the corresponding author on reasonable request.

## Abbreviations

QUIPS: Quality Improvement in Postoperative Pain Management
PROSPECT: Procedure-specific postoperative pain management
PROs: Patient-reported outcomes
POD1: Postoperative Day 1
Total-Tx: Total thyroidectomy
Hemi-Tx: Hemithyroidectomy
Para-Tx: Parathyroidectomy
ASA: American Society of Anesthesiologists
NRS: Numeric Rating Scale
max: Maximum
min: Minimum
n: Number of cases
SD: Standard deviation
ERAS: Enhanced Recovery After Surgery
